# Effect of Buckwheat Groats Processing on the Content and Bioaccessibility of Selected Minerals

**DOI:** 10.3390/foods9060832

**Published:** 2020-06-25

**Authors:** Joanna Klepacka, Agnieszka Najda, Kamila Klimek

**Affiliations:** 1 Department of Commodity Science and Food Analysis, Faculty of Food Science, University of Warmia and Mazury in Olsztyn, Oczapowskiego 2, 10-719 Olsztyn, Poland; klepak@uwm.edu.pl; 2 Laboratory of Quality of Vegetables and Medicinal Plants, Department of Vegetable Crops and Medicinal Plants, University of Life Sciences in Lublin, Akademicka 15, 20-950 Lublin, Poland; 3 Department of Applied Mathematics and Informatics, University of Life Sciences in Lublin, Głęboka 28, 20-612 Lublin, Poland; kamila.klimek@up.lublin.pl

**Keywords:** bioaccessibility, minerals, buckwheat, dehulling, roasting, food processing

## Abstract

Adequate supply of minerals in the diet is necessary for the proper functioning of the human body. In recent years gluten-free diet, which rigorous forms may lead to deficiencies of mineral components (especially Mg, Mn, Zn and Cu), is becoming more and more popular. Buckwheat grains do not contain gluten, and their nutritional value is very high. They are often consumed in the form of groats, which are obtained from roasted and dehulled seeds. The purpose of the work was to determine how conducting the buckwheat groats production in industrial conditions affects the content and availability of magnesium, manganese, zinc and copper. The results indicated that husk removal had a particularly adverse effect on the total manganese content and its amount released by enzymatic digestion, whereas it had a positive effect on the post-digestion zinc level by increasing it by nearly half. Hydrothermal processes especially affected the release of analysed elements simulated by the in vitro method, and the extent of changes depended on the processing parameters. It was shown that bioaccessibility of minerals may be increased by treating buckwheat at a lower temperature for a short time, which has a particularly beneficial effect on the manganese and magnesium. Treating grains at a higher temperature reduces the bioaccessibility of all analysed elements, which was particularly noted for zinc and copper. Based on the obtained results, it should be stated that buckwheat groats should be a regular part of human diet, because they are a good source of easily digestible mineral compounds. Their consumption should be especially considered by people on a rigorous gluten-free diet, as they can prevent mineral deficiencies associated with its use.

## 1. Introduction

Minerals, both macro- and micronutrients, have many important functions in the human body. Their excessively small or large amount in the diet may lead to characteristic disorders and increases the risk of the “diseases of affluence” (osteoporosis, hypertension, cancer, coronary heart disease, diabetes) [1,2,3,4]. The use of mineral components by the body depends on many factors, which are associated with both the diet composition, the physiological condition of the body, as well as the degree of saturation with the component or its chemical species [5]. The bioavailability portion of a nutrient is usually defined as the amount that is absorbed into the blood after being released from the compounds present in food products [6]. The composition of food influences the availability for intestinal absorption, so it is important to determine the availability of minerals from individual products in the diet [7]. The bioaccessibility of mineral nutrients from plant products is lower than from animal products, and the factors limiting their absorption include compounds contained in plant matrix as oxalates, phytates, polyphenolic compounds and dietary fibre, because they bind or chelate metal ions in forms that do not dissolve easily [8].

Much attention has been devoted in recent years to gluten-free food, which is consumed not only by patients diagnosed with gluten-related diseases but also by healthy individuals [9,10]. Some people reach for this type of food considering it as a type of popular reducing diet, which is part of a healthy lifestyle. Many studies indicate nutritional deficiencies in people on rigorous gluten-free diets based on such popular raw materials like corn or rice. They mainly concern mineral compounds and among them especially copper, iron, magnesium and zinc [11]. The good source of these elements can be buckwheat grains, which do not contain gluten and, besides mineral compounds, are also a good source of protein with a favourable amino acid composition, lipids rich in unsaturated fatty acids, and saccharides, among which the large amount of resistant starch and fibre is particularly noteworthy [12]. In the food industry, buckwheat grains are most often processed into flour, groats and flakes, but work is still in progress on new products [13]. Extruded and expanded products made entirely or partially from buckwheat can be found on the market, while buckwheat flour is often used to make pasta, bread and cakes [14]. Groats, produced from roasted and dehulled grains, are the most popular products obtained from buckwheat in many European countries and Japan [15]. Unit operations used in processing may affect the bioaccessibility of minerals to varying extent, changing the content of elements, but also causing degradation or formation of absorption inhibitors or stimulants, and the chemical species or salt solubility of a specific element may vary depending on the physical parameters and duration of the process [16]. The use of different methods of buckwheat groats production, using for that purpose different buckwheat grains, causes the nutritional value of groats available on the market to be different. Ikeda et al. [17] reported that the magnesium content in buckwheat groats available in Japan ranged from 1410 to 1890 µg/g, while groats sold in Europe contained this element on the level from 1810 to 2170 µg/g. These differences also concerned manganese, which was found in groats available in Japan in the amount of 7.9 to 25.3 µg/g, while groats sold in Europe contained this element at the level of 11.6 to 14.7 µg/g. Smaller differences concerned zinc and copper, which content in buckwheat groats available on the Japanese and European market was at the following levels: 12.9–24.1 µg/g and 17.8–26.1 µg/g in the case of zinc and, respectively, 3.1–5.9 and 4.8–6.2 µg/g for copper.

The most important technological operation in the process of buckwheat groats production that affects the bioaccessibility of minerals may be dehulling and consequent removal of substances hindering the use of minerals by the human body. The outer layers of buckwheat grains contain several times more fibre and phytic acid than the inner layers, and the changes in the content of minerals caused by dehulling may also be a consequence of their specific distribution in individual anatomical parts of caryopses [13]. Roasting also affects the compounds that hinder the use of mineral nutrients. Dziedzic et al. [18] and Górecka et al. [19] indicated that this operation causes quantitative changes in different fibre fractions and that the effect of high temperatures also inactivates endogenous phytase, which prevents the decomposition of phytates and the release of minerals. Not all studies confirm the negative impact of hydrothermal treatment on bioaccessibility of minerals, and the factors that affect the results may include the process parameters, such as time, temperature and humidity [11,20].

The study of nutrients bioaccessibility in natural conditions is very difficult, which is definitely affected by the difficult access to the human gastrointestinal tract, especially to the small intestine. This contributed to the rapid development of simpler and cheaper methods of research conducted on in vitro models, which simulate the human digestive system [8].

Since most of the literature data concerns the transformations that take place during buckwheat seed processing in a laboratory, the aim of this study was to determine how the content and bioaccessibility of selected mineral compounds (magnesium, manganese, zinc and copper) is affected by the processes of buckwheat groats production under industrial conditions. The results may be used to identify the factors with positive and negative impact on the availability of analysed compounds, which may be used in planning the buckwheat groats production process to maximize it as a source of minerals in the human diet.

## 2. Materials and Methods

### 2.1. Food Samples

**Figure 1:**
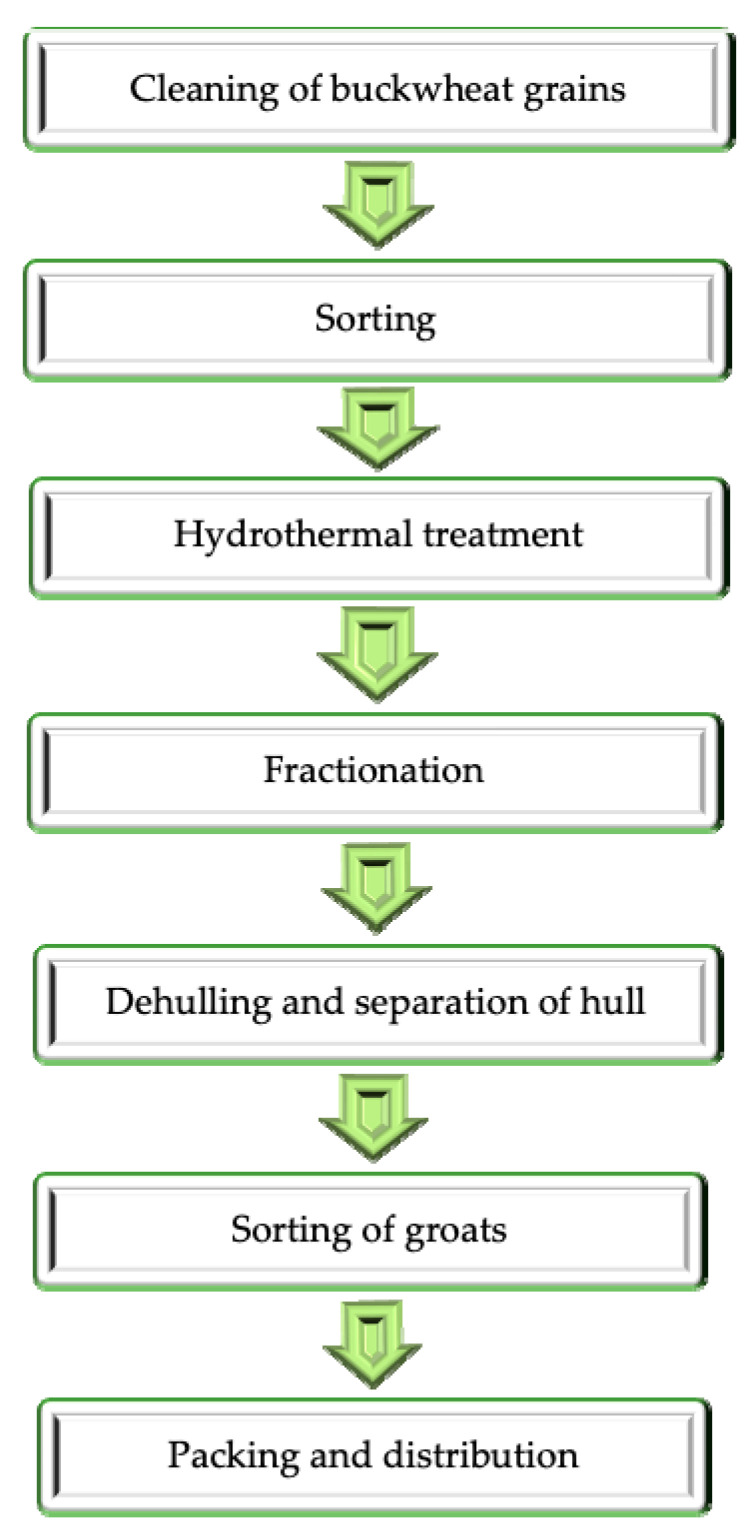
The scheme of buckwheat groats processing.

The study material consisted of samples of buckwheat grains with hull and groats obtained from them, collected three times within a short time interval (up to 2 weeks) from a plant located in Poland, which has been processing buckwheat seeds for many years. Buckwheat grains used as a raw material for groats production were not defined in terms of variety, because seed suppliers do not grow varietally uniform material. The requirements of the plant do not include the varietal purity of buckwheat seeds but only their largest size and the absence of impurities and broken grains. The stages of buckwheat groats production are shown in Figure 1.

Unroasted and roasted groats were used in the study. Hydrothermal treatment of the former was carried out using a “Pedrotti” grain dryer, in which the buckwheat seeds were blown over with air heated to 40–110 °C, and this process was carried out until the seeds moisture content reached 14%, which usually lasted about 10–20 min. Grains were roasted in a pressure boiler with steam as the heating agent and the following seed processing parameter: pressure 0.4–0.45 MPa, temperature 140 °C, time 1.5 h.

The samples collected three times were mixed and homogenized; buckwheat seeds were dehulled by hand, and then, all of the analysed products (grains with hull, dehulled grains and groats) were crushed in an IKA A10 laboratory mill and tested. All analyses were performed in three parallel replicates.

### 2.2. Determination of Minerals

The samples of buckwheat products (2 g) were wet-ashed in a mixture of nitric and perchloric acids (20 mL; 3:1; Suprapure, Merck, Germany) on an aluminium electric heating block (VELP, Milano, Italy) fitted with a programmable temperature setting, increasing the temperature gradually to 200 °C which lasted about 2 h. The colourless mineralisate was transferred to 50 mL volumetric flasks and made up with deionised water to mark. Reagent samples were prepared at the same time. The contents of individual minerals (magnesium, manganese, zinc and copper) were determined by flame atomic absorption spectrometry (acetylene—air flame) with the iCE 3000 Series Atomic Absorption Spectrometer (Thermo-Scientific, Waltham, MA, USA) with a Glite data station, background correction (deuterium lamp) and appropriate cathode lamps [21]. The selected elements determination was performed at the following wavelengths: 285.2 nm (Mg), 279.5 nm (Mn), 213.9 nm (Zn) and 324.8 nm (Cu). The method was validated by a simultaneous analysis of reference material (INCT-MPH-2, mixed polish herbs) with the accuracy for Mg, Mn, Zn and Cu of 99.7%, 99.6%, 101.2% and 98.3%, respectively.

### 2.3. Enzymatic Digestion

The in vitro method of enzymatic digestion of Ikeda [22] with modification as reported by Skibniewska et al. [23] and Nalepa et al. [24] was used. A sample of 5 g of ground products and 50 mL deionized water was adjusted to pH 2 with 1 M HCl (Suprapure, Merck, Germany), then a pepsin solution (Sigma-Aldrich, St. Louis, MI, USA) of 16 g of enzyme/100 mL solution was added, and the sample was incubated for 2 h at 37 °C with shaking. During this stage, the pH was periodically monitored and, if necessary, adjusted by adding 6 M HCl (Suprapure, Merck, Germany). After 2 h, the pH of the solution was raised to 6.8–7 by adding 6% NaHCO_3_ solution (Merck, Germany), and a pancreatin solution (Sigma-Aldrich, USA) was added (0.4 g enzyme/100 mL of 0.1 M NaHCO_3_). The samples were incubated for 4 h at 37 °C with shaking (200 rpm), then centrifuged (4000 rpm for 20 min), filtered and mineralized in a mixture of nitric and perchloric acids (3:1) (Suprapure, Merck, Germany). Determination of the mineral compounds was performed by flame atomic absorption spectrometry in the conditions described above.

The content of mineral compounds released by enzymatic digestion was also expressed in relation to their total content; it was defined as bioaccessibility and expressed in % according to the following formula:

(1) $$\frac{\mathrm{amount}\;\mathrm{of}\;\mathrm{mineral}\;\mathrm{compounds}\;\mathrm{released}\;\mathrm{by}\;\mathrm{enzymatic}\;\mathrm{digestion}\;[\mu g/g]}{\mathrm{total}\;\mathrm{amount}\;\mathrm{of}\;\mathrm{mineral}\;\mathrm{compounds}\;\mathrm{determined}\;\mathrm{in}\;\mathrm{the}\;\mathrm{fresh}\;\mathrm{products}\;(\mathrm{before}\;\mathrm{enzymatic}\;\mathrm{digestion}\;)\;[\mu g/g]}\times 100$$

### 2.4. Statistical Analysis

The present data are mean values of three replicate measurements. The results were analysed statistically with a one-way analysis of variance using the Tukey’s HSD test (Tukey’s honest significant difference test). The normality of the distribution was checked using Shapiro–Wilk test. The inference was performed at the significance level <0.05 and degrees of freedom = 3. A multidimensional data analysis was also carried out using principal component analysis (PCA), which aimed at forming homogeneous groups in such a way that objects with similar parameters were gathered in one cluster. All statistical analyses were performed using SAS Enterprise Guide 5.1 software (SAS Institute Inc., Cary, NC, USA).

## 3. Results and Discussion

### 3.1. The Total Content of Minerals in Buckwheat Grains and Groats

The assay results showed that the analysed raw materials and buckwheat products contained magnesium in the highest amounts and copper in the lowest (their content, depending on the type of the examined product, ranged from 1375.0–1653.1 µg/g and 4.75–6.22 µg/g, respectively (Table 1). The only elements whose content in the same type of products was statistically the same were manganese and zinc determined in grains with hull at the level of 26.38 and 22.30 µg/g, respectively. A similar content of manganese, zinc and copper was found in buckwheat grains by Steadman et al. [25] who reported that the magnesium content was nearly twice as high. Ekholm et al. [26] found the level of this element, as well as manganese and copper, to be similar to that determined in the current study, and they determined the level of zinc to be slightly lower. Three times lower magnesium levels were found by Peng et al. [27], while they reported the content of other minerals within the range shown in the current study. Differences in the content of mineral compounds present in buckwheat grains, as reported by different authors, may be caused by many factors.

Pongrac et al. [20], Lintschinger et al. [28] and Khan et al. [29] pointed to the high impact of environmental factors. The content of mineral components is also affected by the time of seed harvest, but most studies indicate the importance of the species and variety of raw material. Huang et al. [30] studied the concentration of selected minerals in seeds of 123 Tartary buckwheat accessions from the same cultivation. The samples showed high variability of the mineral content, and they found that the average concentration of magnesium was 1523.89 µg/g (with a range of 729–3104 µg/g), zinc content was 27.41 µg/g (with a range of 8.44–66.63 µg/g), and the content of copper was 19.49 µg/g (with a range of 5.74–36.01 µg/g). Trends in the different mineral content of different buckwheat varieties were also emphasised by Unal et al. [31], who reported that the levels of certain elements may differ by a factor of 40.

A general analysis of the changes of mineral compounds caused by buckwheat groats production was made comparing their average content determined in grains and groats, regardless of their type (Table 1). This comparison revealed that only the manganese and magnesium content changed (from 20.28 to 12.56 µg/g and from 1523.1 to 1450.0, respectively), while the level of the other elements did not change during the production of buckwheat groats. Since two processes (dehulling and hydrothermal treatment) are essential in buckwheat grains processing, it seemed interesting to determine how the contents of the elements are affected by each of these processes separately. Buckwheat grains with hulls are roasted under industrial conditions, and only after this process, they are dehulled. Therefore, in order to determine the impact of hull removing process, grains with hull were compared with those dehulled manually in a laboratory. Such a comparison showed that dehulling process influenced the content of most of the analysed components and resulted in an almost twofold reduction of manganese content (from 26.38 to 14.17 µg/g) and an increase of magnesium and zinc content by more than 20% (their amount increased from 1393.1 to 1653.1 µg/g and from 22.30 to 29.30 µg/g, respectively). The observed changes result mainly from the distribution of mineral components in the parts of caryopses. The largest amount of manganese was concentrated in seed hulls, while magnesium and zinc were concentrated in the layers remaining after the hull removal. These trends were confirmed by Pongrac et al. [32], who analysed the distribution of minerals in single buckwheat caryopsis tissues using micro-proton-induced X-ray emission and synchrotron radiation-based low-energy X-ray fluorescence. They found that the concentration of magnesium and zinc in the cotyledon is about 60% of their total amount, 10% in endosperm, about 5% in the aleuronic layer, and their concentration in buckwheat husk is only 15%. They found the content of manganese in buckwheat hull to be almost 40% of its total amount, while its concentration in the cotyledon reached 50%, and in the endosperm and aleuronic layers, it reached 5%. Steadman et al. [33] also confirmed the lower level of magnesium and zinc in the outer layer of the caryopsis. These authors analysed the composition of brans with and without hulls and reported that magnesium and zinc were the most concentrated in bran, particularly bran from which the hulls had been removed, unlike manganese and copper, which were present at higher levels in bran with hull.

**Figure 2:**
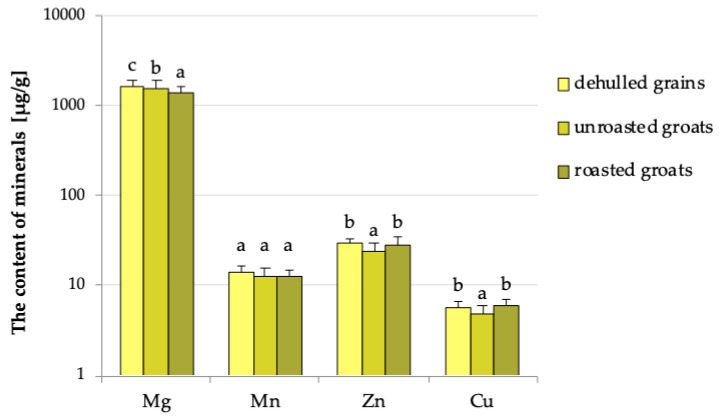
The content of minerals in dehulled buckwheat grains and groats. a, b, c—values referring to individual minerals and denoted by the same letters are not statistically different at *p* < 0.05.

**Figure 3:**
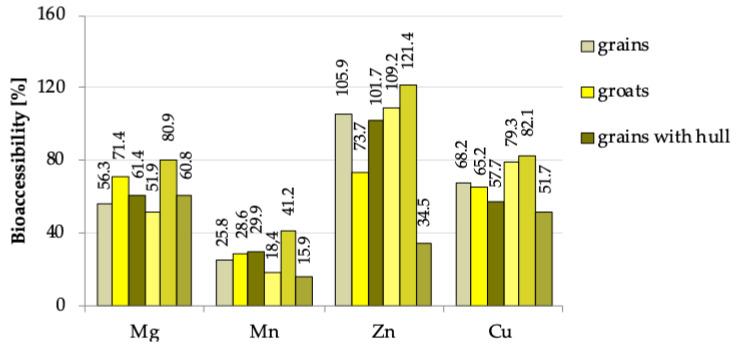
The bioaccessibility [%] of analysed minerals from different types of buckwheat products.

In order to assess the impact of hydrothermal processes without taking into account the changes occurring in the grains during removing their hull, grains dehulled manually were compared with different types of groats (Figure 2 and Figure 3) and also unroasted and roasted groats (third comparison in Table 1 and Table 2). Comparison of dehulled grains with groats is a certain simplification made for the purposes of this work, because in industrial conditions grains with hull are directed to dryers and boilers, and first they are heated and then dehulled (so in the groats taken for analysis not only thermal processes but also mechanical husk removal took place). This comparison eliminates the influence of dehulling process, thanks to which the changes that occur only during the heating process are analysed. This is a very large simplification resulting from the kind of method used in the industrial buckwheat grains treatment.

Based on the comparison presented in Table 1 and Figure 2, it was shown that hydrothermal treatment had the greatest impact on magnesium content by reducing it from 1653.1 µg/g found in dehulled grains to 1525.0 µg/g in unroasted groats and 1375.0 µg/g in roasted groats. A detailed analysis of the data presented in Figure 2 showed that short hydrothermal treatment carried out at a lower temperature caused a statistically significant decrease in the content of most analysed elements, whereas the roasting process carried out at a higher temperature decreased only the magnesium level. A short hydrothermal treatment reduced the zinc content from 29.30 µg/g in grains with hull to 23.73 µg/g in unroasted groats. Such treatment reduced the copper content to a lesser extent; dehulled buckwheat grains and unroasted groats contained the element at 5.79 and 4.75 µg/g, respectively. The hydrothermal treatment did not affect the manganese level, and its content depended only on the dehulling process.

Opinions on the impact of thermal processes on buckwheat grains are ambiguous. Stempińska et al. [34] claimed that the thermal treatment of buckwheat grains did not significantly affect the total mineral content. Similar trends were also demonstrated by Steadman et al. [25] who analysed changes in the total ash content in groats from three producers and showed that its level in unroasted groats ranged from 1.97% to 2.09% and from 1.87% to 2.10% after roasting. Manthey and Hall [35] reported that the content of mineral compounds varied depending on the type of thermally treated product. They analysed the effect of processing and cooking on the ash content and mineral composition of spaghetti fortified with buckwheat bran flour. Their results indicated that extrusion and drying temperature did not affect the mineral composition of spaghetti, but cooking reduced ash content by 28%, copper by 45% and zinc by 11%. Cooking had little effect on the content of calcium, iron, magnesium and manganese. Starzyńska-Janiszewska et al. [36] reported that boiling increased the mineral content in unroasted groats and decreased it in roasted groats, which was also confirmed by Steadman et al. [25,33]. The impact of hydrothermal treatment on the content of mineral compounds was also demonstrated by Florkiewicz et al. [37]. They reported that depending on how buckwheat groats were boiled, their mineral content varied, and the extent of changes depended on both the type of raw material and the processing method. Boiling of roasted buckwheat groats increased the magnesium content, while its level in various types of unroasted groats changed inconclusively. In one of them, due to the extension of seed processing time, the content of this element was found to decrease gradually, while in the other, the content of magnesium initially increased and then decreased. A decrease in the content of this element (and other mineral components) due to thermal processing of buckwheat seeds was also reported by Mota et al. [38]. They assessed the impact of two cooking methods, steaming and boiling, and showed that, regardless of how the seeds are heated, the magnesium and zinc levels decreased by 13% as a result of boiling, while the copper content decreased by 11%, and the manganese content decreased by 6% from their initial levels.

Changes in the content of mineral compounds that occur during heating depend on the used cooking technique, the amount of used water and the degree of grinding of the raw material, as well as whether the mineral components are in a more or less soluble form. During food processing, minerals can be released from complexes with organic compounds, which can change their biological activity and increase their content. Changes in the content of minerals in cooked products can be caused by leaching (dissolving) and passing them to a decoction. Worobiej et al. [39] reported that in cooked buckwheat groats, especially cooked with pouring out water, the amount of ash has decreased as a result of the release of water-soluble compounds. A more pronounced reduction was observed in roasted groats, which the authors explain by the fact that the roasting process reduced the amount of insoluble fibre fraction, which could facilitate the solubility of some compounds. The effect of varying degrees of heat treatment on changes in the content of minerals found in buckwheat seeds was also observed by Deng et al. [40] and Mota et al. [38] who added that the range of observed changes also depends on the type of the grain.

### 3.2. The Content of Minerals Released by Enzymatic Digestion and Their Bioaccessibility from Buckwheat Grains and Groats

An analysis of the content of mineral compounds released by enzymatic digestion showed that, like their total content, it was diverse and depended on technological processes applied in buckwheat seed processing (Table 2). In all samples digested in vitro, the content of magnesium was the highest and that of copper and manganese was the lowest. A comparison of buckwheat grains with groats obtained from them (without classifying them into types) showed that the applied technological process caused a statistically significant increase in the content of magnesium (from 857.6 to 1034.9 µg/g) and a decrease in the content of zinc (from 27.34 to 19.39 µg/g) released by enzymatic digestion. The availability of zinc was also affected by dehulling process, which increased it by nearly half. Hull removal also decreased the level of manganese released by enzymatic digestion by more than half, and it increased the level of copper to a lesser extent, which changed in the opposite direction after the hydrothermal treatment.

Hydrothermal processes also affected the zinc and magnesium levels to a large extent, bringing about (as in the case of copper) changes opposite to those caused by dehulling process. A short hydrothermal treatment almost doubled the content of manganese released by enzymatic digestion (from 2.61 µg/g found in dehulled grains to 5.19 µg/g in unroasted groats), whereas its level decreased under stricter process parameters (to 1.99 µg/g in roasted groats). The zinc content found in the samples after digestion was changed slightly by a short hydrothermal treatment, whereas the roasting process resulted in an almost threefold decrease of its content (from 31.99 µg/g in dehulled grains to 9.97 µg/g in roasted groats) (Table 2).

The content of mineral compounds released by enzymatic digestion was also expressed in relation to the total content of individual mineral components (determined in the fresh products); it was defined as bioaccessibility (Figure 3). Its analysis showed tendencies similar to the relationships discussed above regarding the amount of minerals released after enzymatic digestion (expressed in µg/g). The results confirmed that a shorter hydrothermal treatment carried out at a lower temperature is definitely more beneficial for accessibility of all analysed compounds, compared to the roasting process carried out at a higher temperature and four times longer. Analysing the data shown in Figure 3, it should be stated that dehulling process decreased the bioaccessibility of magnesium and manganese and increased the availability of zinc and copper, while hydrothermal processes had a significant effect on the bioaccessibility of all analysed elements. The short hydrothermal treatment applied during the production of unroasted groats increased the bioaccessibility of all minerals, and the changes were the highest for manganese and magnesium. The roasting process caused the greatest changes in the availability of zinc, which confirms the relationships discussed in the characterisation of data presented in Table 1. The values which determine the bioaccessibility of zinc seem interesting because in most of the analysed buckwheat products they exceed 100%. This is a consequence of the fact that a higher level of this nutrient was found in the digested samples compared to those non-digested in which the total content of this element was determined. After enzymatic digestion, clear supernatant was collected containing zinc released by hydrolysis with pepsin and pancreatin. Pepsin was obtained from porcine stomach mucosa and pancreatin was obtained from porcine pancreas. The biological material, apart from its enzymatic activity, could also exhibit other chemical effects on the nutrients present in buckwheat products, which could lead to their mutual interactions, as a result of which more zinc was released to the supernatant fraction than the sum of its amount released from buckwheat products and present in enzymes in the proper samples. Such high bioavailability of zinc may also result from hydrothermal changes in the properties of oxalates, phytates, polyphenolic compounds and dietary fibre, which bind this mineral compound. Changes in their structure under the influence of heating and enzymatic digestion may cause additional release of zinc, which results in higher bioavailability of this element in digested samples. It is also important to note the possibility of making mistakes and analytical difficulties associated with determination of components present in small quantities and of many factors that affect the results [41]. This notwithstanding, it should be noted that analysed buckwheat products contained high levels of zinc, magnesium and copper available for absorption and a low level of manganese. The high bioaccessibility of zinc is particularly noteworthy because the deficiency of this element influences the concentrations of many minerals such as Fe, Mn, Cu, Mg, Ca, Na and K, which can cause a number of serious diseases [4,6].

**Figure 4:**
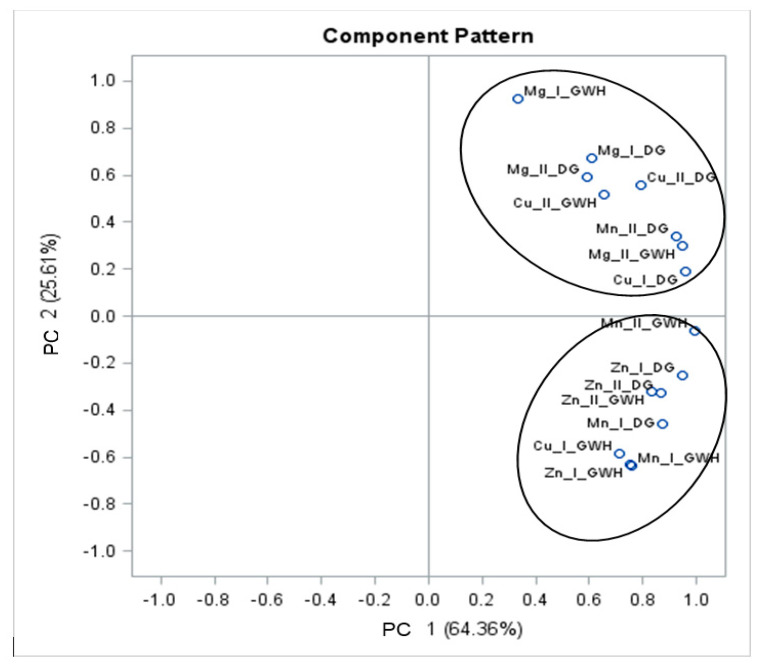
Principal component analysis in assessment of total content of analysed minerals and their amount released by in vitro digestion of buckwheat grains. Explanatory notes: Mg I, Mn I, Zn I, Cu I—total content of minerals in buckwheat grains; Mg II, Mn II, Zn II, Cu II—content of minerals released by in vitro digestion of buckwheat grains; GWH—grains with hull; DG—dehulled grains.

The relationship between the total content of mineral compounds present in buckwheat grains and groats and their amount released by enzymatic digestion was also confirmed by the PCA analysis. It allows multi-dimensional observations to be presented using a small number of coordinates and also allows for the identification of samples differing from other samples in chemical composition. Figure 4 shows the relationship determined for buckwheat grains, which formed two clusters. In the upper right-hand corner of the graph, there are samples of grains with hulls, in which the amount of magnesium released by enzymatic digestion is strongly correlated with the total content of this element in the raw material. Similar relationships were shown in relation to the level of copper and magnesium released by enzymatic digestion from dehulled grains, while in the bottom right-hand corner of the graph there are samples of grains with hull in which the amount of released zinc and manganese depends on the content of these elements in dehulled grains, but these correlations are not strong.

The PCA results allow to look at the entire data structure, but this time not from the separation’s point of view, as in the discriminant analysis, but in order to compare the total content of mineral compounds in buckwheat grains and groats. To achieve this goal, the data space was reduced to two dimensions and the number of main ingredients was selected based on the Kaiser criterion [42]. Together, these two specific components explained more than 87% of the total system variance. The large variation in factor load values allows easy interpretation of individual components, among which PC1 determines the total content of minerals, while PC2 is most strongly associated with their availability and dehulling process (Figure 4).

**Figure 5:**
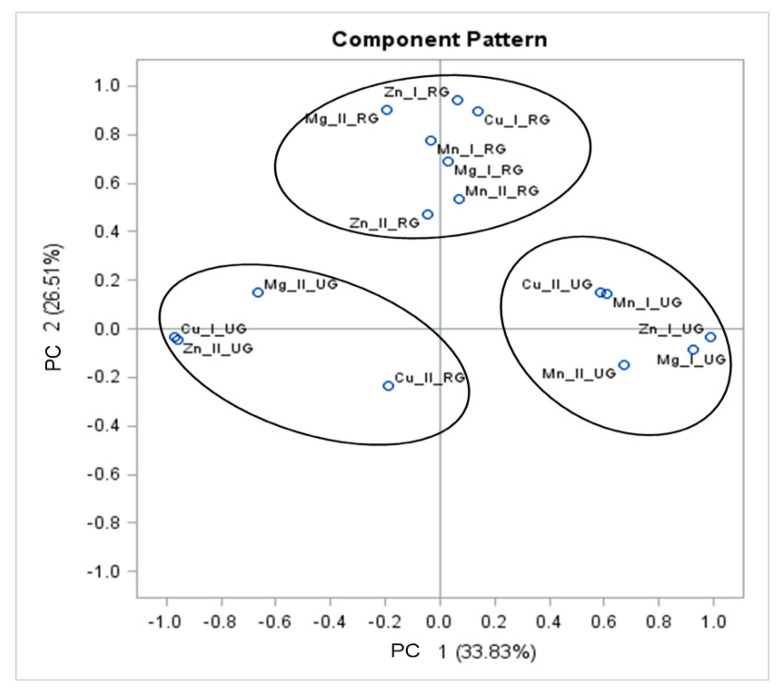
Principal component analysis in assessment of total content of analysed minerals and their amount released by in vitro digestion of buckwheat groats. Explanatory notes: Mg I, Mn I, Zn I, Cu I—total content of minerals in buckwheat groats; Mg II, Mn II, Zn II, Cu II—content of minerals released by in vitro digestion of buckwheat groats; RG—roasted groats; UG—unroasted groats.

The relationship between the amount of the elements released by enzymatic digestion of groats subjected to various hydrothermal treatments and their content before digestion is shown in Figure 5. The variables PC1 and PC2 determined after VARIMAX rotation refer to the following parameters, respectively: PC1 to buckwheat groats heated in different ways, and PC2 to the level of minerals released by enzymatic digestion. Both variables explain more than 60% of system variance. It was found that the level of manganese, magnesium and zinc released by digestion of roasted groats is strongly correlated with their total content, as evidenced by the cluster at the highest values of PC2. In the area of negative values of PC1 and PC2, there was a sample of roasted groats in which the amount of copper released by digestion is inversely correlated with the content of this element in unroasted groats. On the right side of the graph, the third cluster was formed with samples of unroasted groats. This cluster was formed due to the high correlation between the total content of magnesium, zinc, manganese and the level of manganese and copper released by in vitro digestion from the samples of unroasted groats.

Changes in the content and bioaccessibility of the analysed nutrients, caused by the grain treatment processes, probably result to the greatest extent from their interactions with phytate, fibre and phenolic compounds, whose content and chemical structures are affected both by dehulling process and thermal treatment [8,18,19]. Buckwheat grains are a rich source of all of these ingredients, and their influence on the availability of mineral compounds can be different. On the one hand, the high temperature may inactivate endogenous phytase, which prevents the decomposition of phytates and the release of many minerals, while on the other hand, phytates can be broken down during hydrothermal treatment, which releases mineral elements and increases their bioaccessibility [20]. Duliński et al. [43] report that the degree of phytate changes depends on the type and parameters of applied hydrothermal treatment, during which their individual types change to a varying extent. They found that roasting followed by cooking was the most successful combination, resulting in 68% degradation of phytate. These authors also emphasise the effect of the moisture content in the treated products on the reduction of their phytate level. Fairweather-Tait [44] indicates that hydrothermal processes affect the absorption of minerals because an increased temperature and pressure cause the changes in the polysaccharide complexes, which can determine the absorption of minerals in the intestinal lumen. Górecka et al. [19] analysed the effect of technological processes used in buckwheat groats production on the content of dietary fibre and found that the content of neutral fibre, lignin and hemicellulose fractions increased during seed roasting, while the content of cellulose fraction decreased, which significantly affects the availability of mineral compounds. Although these processes may increase the amount of released mineral compounds (which is facilitated by a decrease in the content of insoluble fibre components during roasting), water-soluble components may be removed during roasting. Krishnan and Meera [7] claim that the direction and extent of the changes may vary for different mineral compounds, and such changes depend primarily on the processing type and conditions. The method of seed treatment also significantly affects the content of phenolic compounds, which are strong chelators of many minerals, especially copper and zinc [39,45].

## 4. Conclusions

The content and bioaccessibility of analysed minerals were changed by buckwheat grain treatment processes. The direction of the quantitative changes caused by dehulling process depended mainly on the distribution of analysed minerals in specific seed layers, while the effect of hydrothermal treatment depended on the process parameters, especially the height of the applied temperature. Hull removal has a particularly negative effect on the total content of manganese and its level released by enzymatic digestion, while it has a positive effect on zinc and copper. The total content of analysed components varies slightly under the influence of the thermal treatment, while their bioaccessibility depends significantly on the treatment temperature and its duration. The shorter hydrothermal process carried out at a lower temperature was more beneficial for accessibility of all analysed compounds (especially for manganese and magnesium) than the roasting process carried out longer at a higher temperature (which had especially negative effect on zinc and copper).

The results indicate that buckwheat groats may be a good source of mineral compounds in the daily human diet. Unroasted groats, in particular, should be recommended for people who suffer from mineral deficiencies and especially for those on a gluten-free diet. It should be remembered that the permanent occurrence of mineral deficiencies can seriously disrupt the proper functioning of the body, and because they are exogenous compounds, they should be constantly present in the daily human diet.

## Figures and Tables

**Table 1:** The content of selected minerals in analysed samples depending on the technological process (mean ± SD; values in µg/g).

Type of Process and Product	Type of Analysed Samples	Content of Mineral Elements [µg/g]				*p*-Value
		Mg	Mn	Zn	Cu	
The entire processing (raw material and the final product)	grains ^1^	1523.1 ± 28.22 ^bC^	20.28 ± 1.34 ^bB^	25.80 ± 0.70 ^aB^	6.00 ± 0.24 ^aA^	<0.0001
	groats ^2^	1450.0 ± 31.47 ^aD^	12.56 ± 0.43 ^aB^	26.31 ± 1.66 ^aC^	5.34 ± 0.35 ^aA^	<0.0001
	*p*-value	<0.0001	<0.0001	<0.0100	<0.0100	
Dehulling process	grains with hull	1393.1 ± 27.6 ^aC^	26.38 ± 2.38 ^bB^	22.30 ± 1.02 ^aB^	6.22 ± 0.61 ^aA^	<0.0001
	dehulled grains	1653.1 ± 25.50 ^bD^	14.17 ± 0.77 ^aB^	29.30 ± 0.72 ^bC^	5.79 ± 0.37 ^aA^	<0.0001
	*p*-value	<0.0001	0.0002	<0.0001	0.5533	
Comparison of different methods of hydrothermal treatment	unroasted groats	1525.0 ± 22.2 ^bD^	12.60 ± 0.16 ^aB^	23.73 ± 0.46 ^aC^	4.75 ± 0.09 ^aA^	<0.0001
	roasted groats	1375.0 ± 41.27 ^aD^	12.52 ± 0.67 ^aB^	28.89 ± 2.43 ^bC^	5.92 ± 0.50 ^bA^	<0.0001
	*p*-value	<0.0001	0.6355	0.0492	0.0347	

Data are expressed as mean values ± standard deviations (SDs) of three samples (*n* = 3). The significance of differences was determined using the Tukey test. ^1^ Grains without division into their type (average value calculated for grains with hull and dehulled). ^2^ Groats without division into their type (average value calculated for unroasted and roasted groats). ^a, b^ Values in columns denoted by the same small letters are not statistically different in analysed type of processes at *p* < 0.05. ^A, B, C, D^ Values in rows denoted by the same capital letters are not statistically different between the individual minerals at *p* < 0.05.

**Table 2:** The content of selected minerals released by enzymatic digestion from analysed samples depending on the technological process (mean ± SD; values in µg/g).

Type of Process and Product	Type of Analysed Samples	Content of Mineral Elements Released by In Vitro Digestion [µg/g]				*p*-Value
		Mg	Mn	Zn	Cu	
The entire processing (raw material and the final product)	grains ^1^	857.6 ± 35.02 ^aC^	5.24 ± 0.88 ^aA^	27.34 ± 0.86 ^bB^	4.09 ± 0.11 ^aA^	<0.0001
	groats ^2^	1034.9 ± 47.65 ^bC^	3.59 ± 0.41 ^aA^	19.39 ± 2.37 ^aB^	3.48 ± 0.14 ^aA^	<0.0001
	*p*-value	0.0311	0.1331	<0.0001	0.1500	
Dehulling process	grains with hull	855.7 ± 100.13 ^aD^	7.88 ± 2.20 ^bB^	22.69 ± 0.61 ^aC^	3.59 ± 0.09 ^aA^	<0.0001
	dehulled grains	859.4 ± 19.97 ^aD^	2.61 ± 0.21 ^aA^	31.99 ± 0.72 ^bC^	4.59 ± 0.19 ^bB^	<0.0001
	*p*-value	0.9714	0.0299	<0.0001	0.0002	
Comparison of different methods of hydrothermal treatment	unroasted groats	1233.8 ± 22.4 ^bD^	5.19 ± 0.31 ^bB^	28.81 ± 2.00 ^bC^	3.90 ± 0.46 ^bA^	<0.0001
	roasted groats	836.1 ± 24.3 ^aD^	1.99 ± 0.30 ^aA^	9.97 ± 1.60 ^aC^	3.06 ± 0.13 ^aB^	<0.0001
	*p*-value	0.0169	0.0594	0.0129	0.0602	

Data are expressed as mean values ± standard deviations (SDs) of three samples (*n* = 3). The significance of differences was determined using the Tukey test. ^1^ Grains without division into their type (average value calculated for grains with hull and dehulled). ^2^ Groats without division into their type (average value calculated for unroasted and roasted groats). ^a, b^—Values in columns denoted by the same small letters are not statistically different in analysed type of processes at *p* < 0.05. ^A, B, C, D^—Values in rows denoted by the same capital letters are not statistically different between the individual minerals at *p* < 0.05.

## References

[1] Badimon L., Chagas P., Chiva-Blanch G. (2019). Diet and Cardiovascular Disease: Effects of Foods and Nutrients in Classical and Emerging Cardiovascular Risk Factors. Curr. Med. Chem..

[2] Scherz H., Kirchhoff E. (2006). Trace elements in foods: Zinc contents of raw foods—A comparison of data originating from different geographical regions of the world. J. Food Compos. Anal..

[3] Almohanna H.M., Ahmed A.A., Tsatalis J.P., Tosti A. (2018). The Role of Vitamins and Minerals in Hair Loss: A Review. Dermatol. Ther..

[4] Yu Q., Sun X., Zhao J., Zhao L., Chen Y., Fan L., Li Z., Sun Y., Wang M., Wang F. (2019). The effects of zinc deficiency on homeostasis of twelve minerals and trace elements in the serum, feces, urine and liver of rats. Nutr. Metab..

[5] Lazo A., Lazo P., Urtubia A., Lobos M.G., Gutiérrez C., Hansen H.K. (2019). Copper Analysis by Two Different Procedures of Sequential Extraction after Electrodialytic Remediation of Mine Tailings. Int. J. Environ. Res. Public Health.

[6] Joung H., Nam G., Yoon S., Lee J., Shim J.E., Paik H.-Y. (2004). Bioavailable zinc intake of Korean adults in relation to the phytate content of Korean foods. J. Food Compos. Anal..

[7] Krishnan R., Meera M.S. (2018). Pearl millet minerals: Effect of processing on bioaccessibility. J. Food Sci. Technol..

[8] Suliburska J., Krejpcio Z. (2011). Evaluation of the content and bioaccessibility of iron, zinc, calcium and magnesium from groats, rice, leguminous grains and nuts. J. Food Sci. Technol..

[9] Alsharairi N.A. (2019). Diet and Food Allergy as Risk Factors for Asthma in the Arabian Gulf Region: Current Evidence and Future Research Needs. Int. J. Environ. Res. Public Health.

[10] Rybicka I., Gliszczyńska-Świgło A. (2017). Minerals in grain gluten-free products. The content of calcium, potassium, magnesium, sodium, copper, iron, manganese, and zinc. J. Food Compos. Anal..

[11] Pandey S., Senthil A., Fatema K. (2015). Effect of Hydrothermal Treatment on the Nutritional and Functional Properties of Husked and Dehusked Buckwheat. J. Food Process. Technol..

[12] Ahmed A., Khalid N., Ahmad A., Abbasi N.A., Latif M.S.Z., Randhawa M.A. (2013). Phytochemicals and biofunctional properties of buckwheat: A review. J. Agric. Sci..

[13] Bonafaccia G., Gambelli L., Fabjan N., Kreft I. (2003). Trace elements in flour and bran from common and tartary buckwheat. Food Chem..

[14] Giménez-Bastida J.A., Zieliński H. (2015). Buckwheat as a Functional Food and Its Effects on Health. J. Agric. Food Chem..

[15] Christa K., Soral-Śmietana M. (2008). Buckwheat grains and buckwheat products—Nutritional and prophylactic value of their components—A review. Czech J. Food Sci..

[16] Roy M., Dutta H., Jaganmohan R., Choudhury M., Kumar N., Kumar A. (2019). Effect of steam parboiling and hot soaking treatments on milling yield, physical, physicochemical, bioactive and digestibility properties of buckwheat (*Fagopyrum esculentum* L.). J. Food Sci. Technol..

[17] Ikeda S., Yamashita Y., Kusumoto K., Kreft I. (2005). Nutritional characteristics of minerals in various byckwheat groats. Fagopyrum.

[18] Dziedzic K., Górecka D., Kucharska M., Przybylska B. (2012). Influence of technological process during buckwheat groats production on dietary fibre content and sorption of bile acids. Food Res. Int..

[19] Górecka D., Dziedzic K., Sell S. (2010). The influence of the technological processes applied to production of buckwheat groats on the dietary fiber content. Sci. Nat. Technol..

[20] Pongrac P., Scheers N., Sandberg A.-S., Potisek M., Arčon I., Kreft I., Kump P., Vogel-Mikuš K. (2016). The effects of hydrothermal processing and germination on Fe speciation and Fe bioaccessibility to human intestinal Caco-2 cells in Tartary buckwheat. Food Chem..

[21] Whiteside P.J. (1979). Atomic Absorption, Data Book.

[22] Ikeda S. (1990). Dietary zinc and the zinc components in various foods subjected to in-vitro enzymic digestion. J. Sci. Food Agric..

[23] Skibniewska K., Kozirok W., Fornal L., Markiewicz K. (2002). In vitro availability of minerals from oat products. J. Sci. Food Agric..

[24] Nalepa B., Siemianowska E., Skibniewska K. (2011). Influence of Bifidobacterium bifidum on Release of Minerals from Bread with Differing Bran Content. J. Toxicol. Environ. Health Part A.

[25] Steadman K.J., Burgoon M.S., Lewis B.A., Edwardson S.E., Obendorf R.L. (2001). Minerals, phytic acid, tannin and rutin in buckwheat seed milling fractions. J. Sci. Food Agric..

[26] Ekholm P.J., Reinivuo H., Mattila P.H., Pakkala H., Koponen J., Happonen A., Hellström J., Ovaskainen M.-L. (2007). Changes in the mineral and trace element contents of cereals, fruits and vegetables in Finland. J. Food Compos. Anal..

[27] Peng L.-X., Huang Y.-F., Liu Y., Zhang Z.-F., Lu L.-Y., Zhao G. (2014). Evaluation of Essential and Toxic Element Concentrations in Buckwheat by Experimental and Chemometric Approaches. J. Integr. Agric..

[28] Lintschinger J., Fuchs N., Moser H., Jager R., Hlebeina T., Markolin G., Gossler W. (1997). Uptake of various trace elements during germination of wheat, buckwheat and quinoa. Plant Foods Hum. Nutr..

[29] Khan F., Arif M., Khan T.U., Khan M.I., Bangash J.A. (2013). Nutritional evaluation of common buckwheat of four different villages of Gilgit-Baltistan. J. Agric. Biol. Sci..

[30] Huang X.-Y., Zeller F.J., Huang K.-F., Shi T.-X., Chen Q.-F. (2013). Variation of major minerals and trace elements in seeds of tartary buckwheat (*Fagopyrum tataricum* Gaertn.). Genet. Resour. Crop. Evol..

[31] Unal H., Izli G., Izli N., Asik B.B. (2016). Comparison of some physical and chemical characteristics of buckwheat (Fagopyrum esculentumMoench) grains. CyTA J. Food.

[32] Pongrac P., Vogel-Mikuš K., Jeromel L., Vavpetič P., Pelicon P., Kaulich B., Gianoncelli A., Eichert D., Regvar M., Kreft I. (2013). Spatially resolved distributions of the mineral elements in the grain of tartary buckwheat (*Fagopyrum tataricum*). Food Res. Int..

[33] Steadman K.J., Burgoon M., Lewis B., Edwardson S., Obendorf R. (2001). Buckwheat Seed Milling Fractions: Description, Macronutrient Composition and Dietary Fibre. J. Cereal Sci..

[34] Stempińska K., Soral-Śmietana M., Zieliński H., Michalska A. (2007). Effect of thermal treatment on chemical and antioxidant properties of buckwheat grains. Food Sci. Technol. Qual..

[35] Manthey F.A., Hall C. (2007). Effect of processing and cooking on the content of minerals and protein in pasta containing buckwheat bran flour. J. Sci. Food Agric..

[36] Janiszewska A.S., Stodolak B., Duliński R., Bączkowicz M., Mickowska B., Wikiera A., Byczyński Ł. (2015). Effect of Solid-State Fermentation Tempe Type on Antioxidant and Nutritional Parameters of Buckwheat Groats as Compared with Hydrothermal Processing. J. Food Process. Preserv..

[37] Florkiewicz A., Filipiak-Florkiewicz A., Topolska K., Cieślik E., Kapusta-Duch J. (2015). The effect of hydrothermal processing on the content of selected minerals in different varietes of groats. J. Health Environ. Res..

[38] Mota C., Nascimento A.C., Santos M., Delgado I., Coelho I., Rego A., Matos A.S., Torres D.P., Castanheira I., Motta C. (2016). The effect of cooking methods on the mineral content of quinoa (*Chenopodium quinoa*), amaranth (*Amaranthus* sp.) and buckwheat (*Fagopyrum esculentum*). J. Food Compos. Anal..

[39] Worobiej E., Piecyk M., Perzyna G., Turos J. (2017). Effect of processing and thermally treating buckwheat grains on nutrients. Food Sci. Technol. Qual..

[40] Deng Y., Padilla-Zakour O., Zhao Y., Tao S. (2015). Influences of high hydrostatic pressure, microwave heating, and boiling on chemical compositions, antinutritional factors, fatty acids, in vitro protein digestibility, and microstructure of buckwheat. Food Bioprocess Technol..

[41] Dos Santos A.M.P., Lima J.S., Anunciação D.S., Souza A.S., Dos Santos D.C.M.B., Matos G.D. (2012). Determination and Evaluation Employing Multivariate Analysis of the Mineral Composition of Broccoli (*Brassica oleracea* L. var. Italica). Food Anal. Methods.

[42] Irwing P., Hughes D.J., Irwing P., Booth T., Hughes D.J. (2018). Test Development. The Wiley Handbook of Psychometric Testing: A Multidisciplinary Reference on Survey, Scale and Test Development.

[43] Duliński R., Janiszewska A.S., Byczyński Ł., Błaszczyk U. (2016). Myo-inositol phosphates profile of buckwheat and quinoa seeds: Effects of hydrothermal processing and solid-state fermentation withRhizopus oligosporus. Int. J. Food Prop..

[44] Fairweather-Tait S.J. (1999). The importance of trace element speciation in nutritional sciences. Anal. Bioanal. Chem..

[45] Zieliński H., Michalska-Ciechanowska A., Piskula M.K., Kozłowska H. (2006). Antioxidants in thermally treated buckwheat groats. Mol. Nutr. Food Res..

